# Diagnosis, Mechanisms, and Differentiation of Inflammatory Diseases of the Outer Retina: Photoreceptoritis versus Choriocapillaritis; A Multimodal Imaging Perspective †

**DOI:** 10.3390/diagnostics12092179

**Published:** 2022-09-09

**Authors:** Ioannis Papasavvas, Alessandro Mantovani, Carl P. Herbort

**Affiliations:** 1 Retinal and Inflammatory Eye Diseases, Centre for Ophthalmic Specialized Care (COS), Rue Charles-Monnard 6, 1003 Lausanne, Switzerland; 2 Department of Ophthalmology, Valduce Hospital, 22100 Como, Italy

**Keywords:** primary photoreceptoritis, primary inflammatory choriocapillaropathies (PICCPs), multiple evanescent white dot syndrome (MEWDS), idiopathic multifocal choroiditis (MFC), indocyanine green angiography (ICGA)

## Abstract

Background and aim: Inflammatory diseases that affect the outer retina do so by different mechanisms. Some of them result from the direct, primary involvement of the outer retina (primary photoreceptoritis) such as acute zonal outer occult retinopathy (AZOOR). Others affect the photoreceptors secondarily due to the inflammatory involvement of the choriocapillaris. This results in choriocapillaris non-perfusion that damages the photoreceptors due to the ensuing ischaemia, a mechanism characterising primary inflammatory choriocapillaropathies (PICCPs) such as multiple evanescent white dot syndrome (MEWDS), idiopathic multifocal choroiditis (MFC), and others. Thanks to multimodal imaging (MMI), it is now possible to differentiate between these two mechanisms of outer retinal damage. The aim of this study is to determine the MMI characteristics that allow us to differentiate primary photoreceptoritis, including AZOOR, from PICCPs such as MEWDS and MFC. Methods: A series of eight PICCPs cases (five typical MEWDS and three typical active MFC cases) and four typical primary photoreceptoritis/AZOOR cases (five eyes) that had undergone complete MMI investigation, including fundus photography (FP), blue light fundus autofluorescence (BL-FAF), spectral domain optical coherence tomography (SD-OCT), OCT angiography (OCT-A, when available), fluorescein angiography (FA), and indocyanine green angiography (ICGA) were analysed, pointing out the differences that allow us to distinguish primary photoreceptoritis from PICCPs. Results: All primary photoreceptoritis/AZOOR cases showed (1) faint fundus pallor around the fovea, (2) BL-FAF hyperautofluorescence, (3) loss of photoreceptor outer segments (PROS) on SD-OCT, (4) absence of choriocapillary drop-out on OCT-A, (5) normal FA or faint FA hyperfluorescence, and (6) conserved ICGA fluorescence/no hypofluorescent areas; (1), (2), (3), and (5) indicated loss of photoreceptor outer segments, and (4) and (6) indicated conserved choriocapillaris circulation. For PICCPs, (a) fundus showed discreet white dots or none (in MEWDS) and punched-out scars in MFC, (b) BL-FAF hyperautofluorescence, (c) loss of PROS on SD-OCT, (d) FA faint hyperfluorescence in MEWDS, also minimal in active MFC lesions (e) in all cases ICGA hypofluorescent areas; (b) and (c) indicating loss of PROS, and (e) indicating choriocapillaris non-perfusion in all cases. The OCT-A did not show consistent findings with faint or no capillary drop-out in MEWDS and MFC. Conclusions: MMI combining the SD-OCT and BL-FAF clearly showed loss of PROS in both groups, while the ICGA determined whether this was due to choriocapillaris non-perfusion in PICCPs or whether the choriocapillaris was intact in case of primary photoreceptoritis. The FA and OCT-A were found to be less useful and/or less sensitive for the appraisal of both these entities.

## 1. Introduction

The outer retina is a watershed region depending mostly on the choriocapillaris for its supply of oxygen and nutrients rather than from retinal circulation. Furthermore, structures like the photoreceptors are very vulnerable and easily damaged by hypoxemia, as these cells are metabolically very demanding [1]. A lack of oxygen, as occurs in inflammatory choriocapillaris non-perfusion, can lead to loss of the outer segments of photoreceptors [1]. This is in contrast with the retinal pigment epithelium (RPE) cells that are metabolically much more resistant than most other cells to oxidative stress, as well as hypoxemic insult [2]. Moreover, Kurihara et al. further showed experimentally in rats that RPE cells doubled in size when susceptible to hypoxia, starting to stock more nutrients to survive, depriving the photoreceptors of badly needed energy, explaining the deleterious effects on photoreceptors [1].

Inflammatory diseases producing damage to the outer retina have been characterised with difficulty in the past. Their classification was not clear before multimodal imaging (MMI) became available and could better identify the structures involved and the sequence of events.

At least two main types of lesion processes can be identified. On one side, photoreceptors can be damaged secondarily, the main mechanism for this being inflammatory choriocapillaris non-perfusion (choriocapillaritis) producing ischaemia on the RPE-photoreceptor complex, as explained hereabove. The gold standard to detect even minimal choriocapillaris non-perfusion and characterise primary inflammatory choriocapillaropathies (PICCPs) is indocyanine green angiography (ICGA) [3]. The degree of damage can be very variable from one choriocapillaritis to another. Among choriocapillaritis entities, multiple evanescent white dot syndrome (MEWDS) and idiopathic multifocal choroiditis (MFC) have a similar, moderately severe involvement of the choriocapillaris; this is quite homogenous between cases, and the pathophysiology can therefore be analysed comprehensively. MEWDS produces less ischaemia as it results from end-choriocapillary closure and as a result, photoreceptors recover faster, whereas in MFC the vessel closure is more proximal, and choriocapillary non-perfusion areas are slightly larger and can produce chorioretinal scars due to RPE damage [4]. Other choriocapillaritis entities such as acute posterior multifocal placoid pigment epitheliopathy/acute multifocal choriocapillaritis (APMPPE/AMIC), because of the great variability and diverse severity from one case to another, are less suited to determine the nature of events of the lesion processes by multimodal imaging.

On the other hand, photoreceptors can be damaged primarily and the condition can then be called primary photoreceptoritis. The mechanisms leading to primary photoreceptoritis are much less known and rely mostly on conjectures. Acute zonal outer occult retinopathy (AZOOR) is one condition causing primary photoreceptoritis [5]. Inflammatory factors certainly play a role, as the efficacy of corticosteroids has been reported in treatment [6]. A degenerative hypothesis has also been put forward, as anti-retinal antibodies have been detected in some cases [7].

In the past, it was not always possible to determine the pathophysiological process in diseases of the outer retina, and choriocapillaritis as well as primary photoreceptoritis were entities classified together by several authors, including Gass, who thought they were variants of the same autoimmune disorder that he called the “AZOOR complex disorders” [8,9,10,11].

Thanks to MMI, it is now possible to distinguish the diverse mechanisms involved in outer retinal disorders and the aim of this case series is to practically demonstrate the contribution of imaging to precisely illustrate, analyse, and classify the inflammatory diseases of the outer retina.

## 2. Patients and Methods

In this retrospective study, patients seen in the Centre for Ophthalmic Specialised care, from 2012 to 2018, with inflammatory outer retinal pathology characterised by damage or loss of photoreceptor outer segments including MEWDS, MFC, and AZOOR-like outer retinopathy, and had been investigated by MMI were included. Patients with low-quality images or with incomplete MMI were excluded. Imaging analysis included fundus photography, fluorescein angiography (FA), indocyanine green angiography (ICGA) spectral domain optical coherence tomography (SD-OCT), blue-light fundus autofluorescence (BL-FAF) (Heidelberg Engineering GmbH, Heidelberg, Germany), and OCT angiography (OCT-A quality above 6/10) (AngioVue^®^, Optovue, Fremont, CA, USA). Best corrected visual acuity (BCVA), visual field recording using the Octopus G program (Octopus, Haag-Streit, Köniz, Switzerland) and/or microperimetry (OTI microperimetry device, OTI, Toronto, ON, Canada) were performed. Investigations were centered only on MMI findings at presentation without details on treatment and evolution.

## 3. Results

### 3.1. Demographics and Functional Data at Presentation

Five patients diagnosed with MEWDS (three men and two women), three MFC patients (all women), and four patients (five eyes) (three women/one man) diagnosed with primary photoreceptoritis were included in the study. The mean age at presentation was 39.4 ± 13.6 years and there was no significant difference in age between the two analysed conditions. The BCVA (Snellen) at presentation was 0.73 ± 0.28 (standard deviation, SD) in the MEWDS + MFC group, and 0.48 ± 0.41 SD in the photoreceptoritis group (NS). There was no significant difference in BCVA among both groups (*p* = 0.13, Student’s *t*-test). Visual field impairment was very diverse from one patient to another in both groups. The mean of the mean defect (MD) was 5.27 ± 3.9 dB in the MEWDS + MFC group versus 17.64 ± 8.45 dB in primary photoreceptoritis, which was highly statistically different (*p* < 0.004, Student’s *t*-test).

### 3.2. Multimodal Imaging of Cases at Presentation

MEWDS case 1

**Figure 1:**
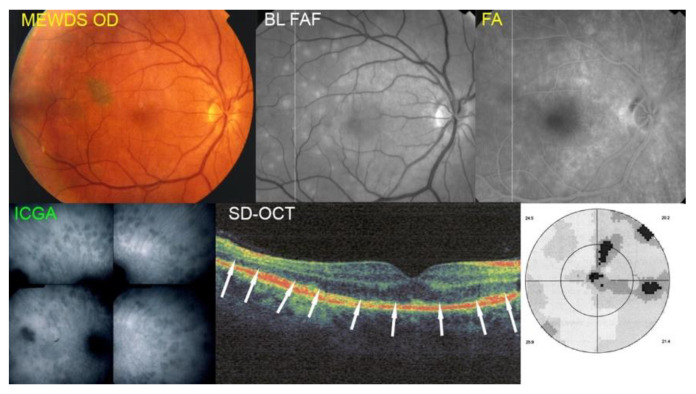
Multimodal Imaging of MEWDS case 1 (see text).

A 56year-old man presented with symptoms of photopsias and subjective visual field loss on the right side. The BCVA was 0.3 and the patient presented a limited visual field impairment (MD = 4.8 dB). Multimodal imaging of patient No. 1 is illustrated in Figure 1. Fundus examination showed foveal granularity but no white dots (top left). The BL-FAF showed extensive posterior pole hyperautofluorescence (top middle) that corresponded with numerous ICGA hypofluorescent areas (bottom left) corresponding to photoreceptor outer segment loss on the SD-OCT (bottom middle, white arrows). The FA showed very faint hyperfluorescent areas (top right). The OCT-A was not available for this patient. Clearly, the origin of outer retina lesions resulted from choriocapillaris non-perfusion, as shown by the ICGA hypofluorescent areas.

MEWDS case 2

A 39-year-old man presented with a history of photopsias in his left eye. The BCVA was 1.0 and visual field impairment was moderate with a mean defect (MD) of 4.1 dB.

**Figure 2:**
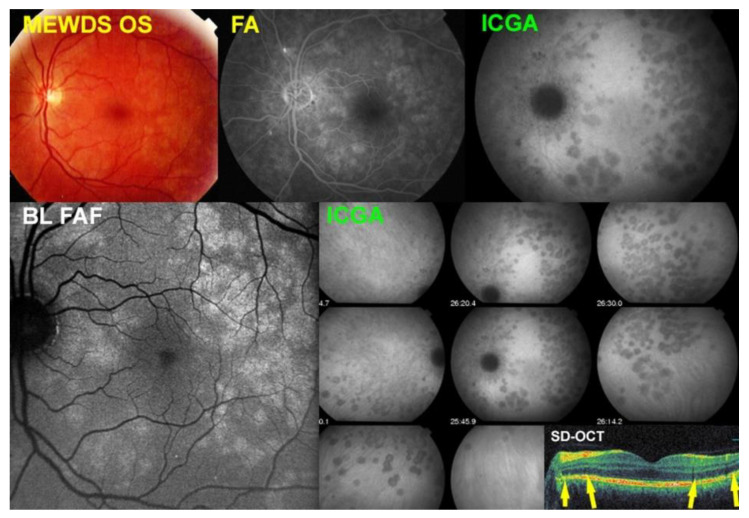
Multimodal Imaging of MEWDS case 2 (see text).

Figure 2 shows the multimodal imaging pictures of MEWDS patient No 2. Fundus photography showed granularity of the fovea but no white dots (top left). The BL-FAF showed numerous areas of hyperautofluorescence in the posterior pole (bottom left) corresponding to extensive areas of choriocapillary non-perfusion of the posterior pole, shown by ICGA (top right) which extended into the mid-periphery (bottom right panoramic frames). The FA (top middle) contributed little to the appraisal showing faint and small hyperfluorescent areas. The SD-OCT (insert bottom right) showed a loss of photoreceptor outer segments in the areas of BL-FAF hyperautofluorescence/ICGA hypofluorescence. No OCT-A was available for this patient.

MEWDS case 3

A 46-year-old woman presented with blurry vision and photopsias. The BCVA was 0.9. Visual field impairment was severe with an extensive centroceacal scotoma with a mean defect (MD) of 13.6 dB.

**Figure 3:**
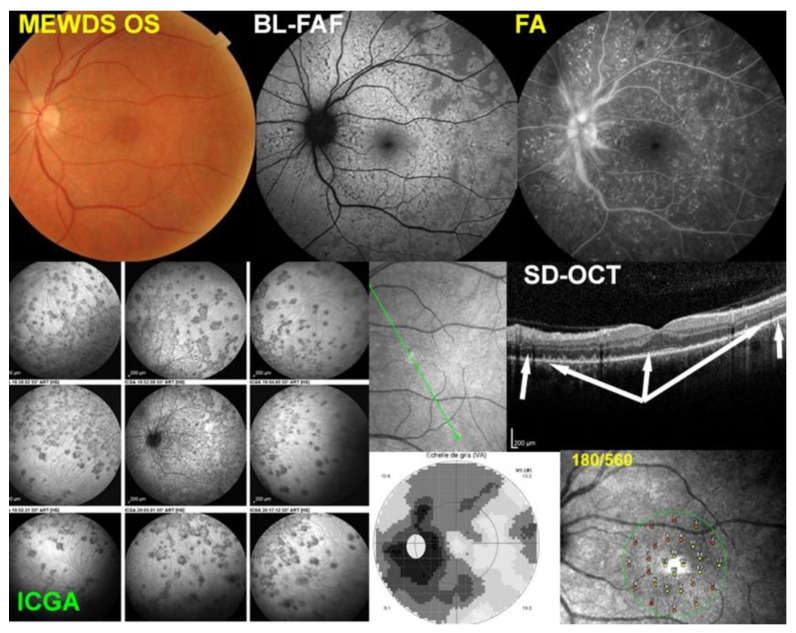
Multimodal Imaging of MEWDS case 3 (see text).

Figure 3 shows the multimodal imaging pictures of MEWDS patient No. 3. The top left fundus picture showed granularity of the fovea without visible white dots. The BL-FAF showed extensive FAF hyperautofluorescence of the posterior pole (top middle) corresponding to numerous ICGA hypofluorescent areas extending much more into the periphery (bottom left). The SD-OCT of the corresponding BL-FAF and ICGA areas showed extensive loss of photoreceptor outer segments (middle right). In this case, the FA showed more extensive areas of hyperfluorescence as well as retinal vasculitis (top middle). The two bottom right frames show loss of visual field on the Octopus® G1 program with an MD of 13.6 dB (bottom middle) and by microperimetry with retinal sensitivity reduced to 180/560 (bottom right). No OCT-A was available for this patient.

MEWDS case 4

**Figure 4:**
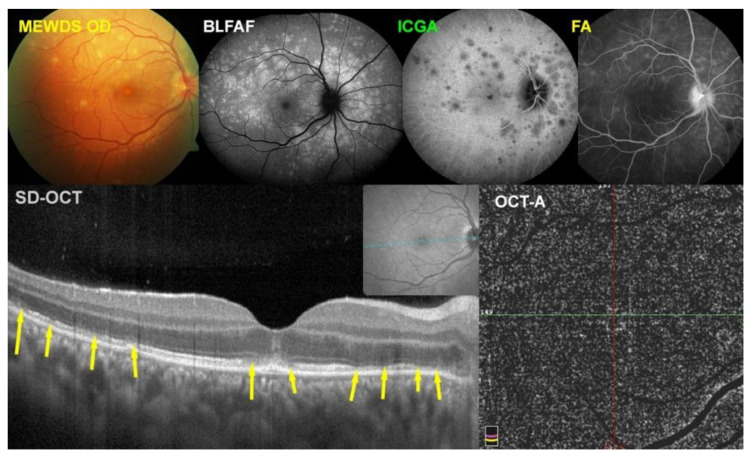
Multimodal Imaging of MEWDS case 4 (see text).

This 31-year-old lady presented typical symptoms of photopsias and subjective visual field disturbance. Figure 4 is illustrating the multimodal imaging of case No. 4. At presentation, her fundus showed the typical fundal white dots (top left) corresponding both to BL-FAF hyperautofluorescent lesions (top middle-left) and ICGA hypofluorescent areas (top middle-right). The SD-OCT (bottom left, yellow arrows) showed zones of loss of photoreceptor outer segments corresponding to BL-FAF hyperautofluorescence and ICGA hypofluorescence. The FA showed minimal signs (top right) and was not contributive to the explanation of the pathophysiology of MEWDS. The OCT-A (bottom right) did not show choriocapillary drop-out because this imaging modality based on flow cannot account for endcapillary non-perfusion in which there is slow or absent flow.

MEWDS case 5

This 27-year-old man presented a fluctuating subjective scotoma in the last 10 days and signaled blurred vision in his right eye. The BCVA was 0.6. There was minimal visual field impairment with a mean defect (MD) of 2.6 dB but a loss of retinal sensitivity on microperimetry, reduced to 320/560.

**Figure 5:**
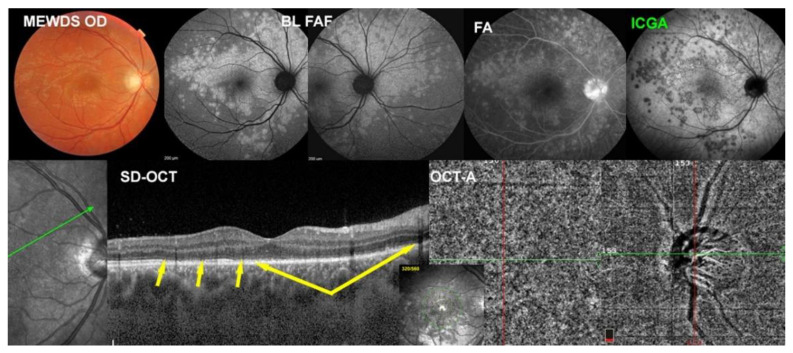
Multimodal Imaging of MEWDS case 5 (see text).

Figure 5 is illustrating the multimodal imaging of case No. 5. Fundus photography (top left) showed granularity of the fovea in the absence of white dots. Extensive areas of bright BL-FAF hyperautofluorescence were present in the posterior pole and nasally (top middle-left frames), co-localising with ICGA hypofluorescent areas (top right). The SD-OCT (bottom left) showed a loss of photoreceptor outer segments in the areas corresponding to FAF hyperautofluorescence and ICGA hypofluorescence, indicating choriocapillaris non-perfusion. The FA showed faint small areas of hyperfluorescence (top middle-right). The OCT-A (bottom right) failed to show choriocapillaris drop-out, as it is unable to image end-capillary low-flow choriocapillary vessels. The insert (bottom-middle) shows reduced retinal sensitivity to 320/560 on microperimetry, while the VF defect was minimal with a normal MD of 2.6 dB (not shown).

MFC case 1

**Figure 6:**
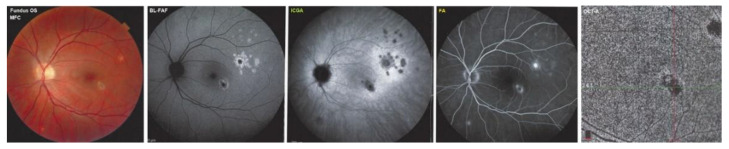
Multimodal Imaging of MFC case 1 (see text).

This female patient, aged 33, had been diagnosed elsewhere with bilateral MFC with photopsias OD. Multimodal imaging of MFC case No. 1 is shown in Figure 6. The BL-FAF hyperautofluorescence and ICGA hypofluorescence dots were present around the right optic disc. On the left, a chorioretinal neovascular membrane (NVM) was noted and had been treated with an intraocular anti-VEGF injection. The patient further received triple immunosuppressive therapy, including 50 mg of prednisone with progressive tapering, mycophenolic acid (Myfortic^®^ 2 × 720 mg), and cyclosporine (150 mg/d). Four months later she complained of photopsias OS with multimodal imaging showing a new episode of MFC OS (Figure 6). The BCVA was 1.0 with no visual field impairment. The fundus examination (Figure 6, far left) showed two areas of chorioretinal changes compatible with neovascular membranes OS, confirmed by FA and ICGA. Around the eccentric NVM, at the end of the temporal superior arcade, there were numerous BL-FAF hyperautofluorescent dots (second from the left) co-localizing with ICGA hypofluorescent dots (middle). The SD-OCT lesions were minimal (not shown) and OCT-A, apart from the two areas of neovascular membranes, did not show additional non-perfusion areas (far right). The FA was non-contributary (second from the right). Another intravitreal injection of an anti-VEGF agent was performed OS and prednisone was re-increased.

MFC case 2

**Figure 7:**
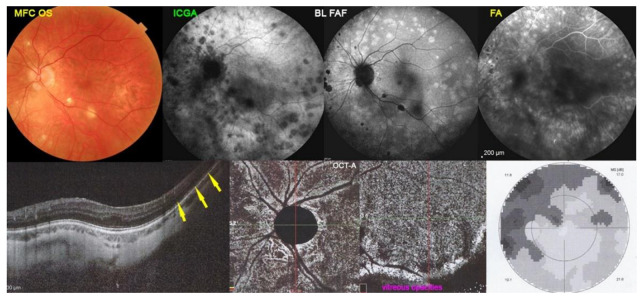
Multimodal Imaging of MFC case 2 (see text).

A 48-year-old woman presented a recurrence of symptomatology OS, including photopsias and an impression of subjective scotomas compatible with a new episode of MFC. Her first episode had been diagnosed as MEWDS OD but one year later a recurrence with punched-out small scars led to re-orient the diagnosis to MFC. This new episode in the contralateral left eye occurred eight years after the previous episode in the right eye. The BCVA OS was 0.7 and there was a moderate visual field impairment with a mean defect (MD) of 7.2 dB. Multimodal imaging is shown in Figure 7. Fundus photography showed small punched-out chorioretinal scars (top left). The ICGA showed many areas of hypofluorescent non-perfused areas (top middle-left), co-localising with areas of BL-FAF hyperautofluorescence (top middle-right). The FA hyperfluorescence showed unusually pronounced hyperfluorescent areas (top right). The SD-OCT showed loss of photoreceptor outer segments corresponding to BL-FAF hyperautofluorescence/ICGA hypofluorescence (bottom left). The OCT-A did not show significant areas of choriocapillary drop-out (bottom-middle pictures). Functionally, visual field showed a substantial scotoma (MD = 7.2 dB) (bottom right).

MFC case 3

**Figure 8:**
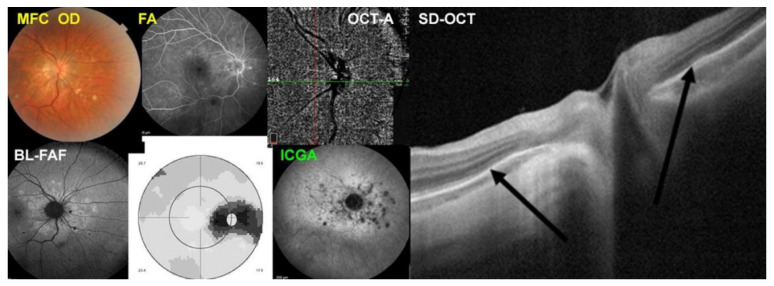
Multimodal Imaging of MFC case 3 (see text).

A 46-year-old woman presented rapidly when she noticed photopsias OD. She was very well aware of the symptomatology, as she had been treated with triple immunosuppressive therapy for MFC in her left eye for three years, seventeen years previously. The BCVA OD was 1.0 but there was a moderate visual field impairment with a mean defect (MD) of 6.0 dB. The presentation was typical for a new episode of MFC. Multimodal imaging of the case is illustrated in Figure 8. Typically, the fundus photography showed small peripapillary punched-out chorioretinal scars (top left), peripapillary BL-FAF hyperautofluorescence (bottom left), and ICGA hypofluorescent dots (bottom middle-right). The SD-OCT (right) showed loss of photoreceptor outer segments in the areas corresponding to BL-FAF hyperautofluorescent/ICGA hypofluorescent areas. The FA showed faint hyperfluorescent areas and did not contribute to the pathophysiological explanation of the MFC process (top middle-left). Visual field Octopus^®^ G1 program showed a scotoma corresponding to the BL-FAF hyperautofluorescent/ICGA hypofluorescent area (bottom middle-left).

**Figure 9:**
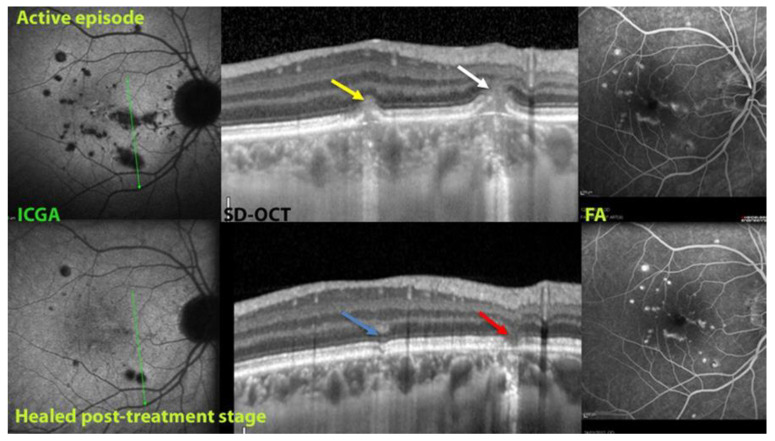
MFC active lesions (upper three images) and results after treatment (bottom three images). Top ICGA image shows hypofluorescent active lesions that disappeared after treatment (bottom ICGA). Top SD-OCT image shows two different active lesions. The first hyperreflective lesion does not interrupt the external limiting membrane (ELM) and affects slightly the IS/OS line (yellow arrow), which is preserved after treatment (blue arrow). On the contrary, the other lesion is more pronounced with disruption of ELM and IS/OS line (white arrow), which led to a scar after treatment (red arrow). FA does not contribute much to the follow-up of the disease (two right images).

In some active MFC cases, SD-OCT presented hyperreflective-cone shape lesions. In case the IS/OS zone was not disrupted by these lesions and the patient was treated properly, these lesions resulted in the maintenance of a healthy IS/OS line (Figure 9, yellow and blue arrow). In other cases, disruption of IS/OS line led to punched-out scars (Figure 9, white and red arrows).

Photoreceptoritis case 1

**Figure 10:**
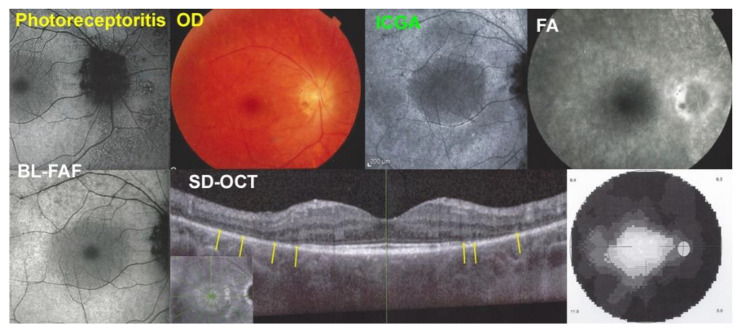
Multimodal Imaging of Photoreceptoritis case 1 (see text).

A 36-year-old woman presented with a decrease in visual acuity and a tubular visual field OD (MD = 19.7 dB). The BCVA was 0.8. Multimodal imaging, illustrated in Figure 10, showed: a fundus picture with a pale halo around the central fovea due to the loss of photoreceptor photopigment (top middle-left), a diffuse BL-FAF hyperautofluorescence of the whole fundus with exception of the central macula (top and bottom left pictures) but no ICGA hypofluorescent areas (top middle-right), and a normal FA (top right). The SD-OCT showed diffuse loss of photoreceptor outer segments except in the central macula (bottom-middle, yellow arrows) explaining the tubular visual field (bottom right).

Photoreceptoritis case 2

**Figure 11:**
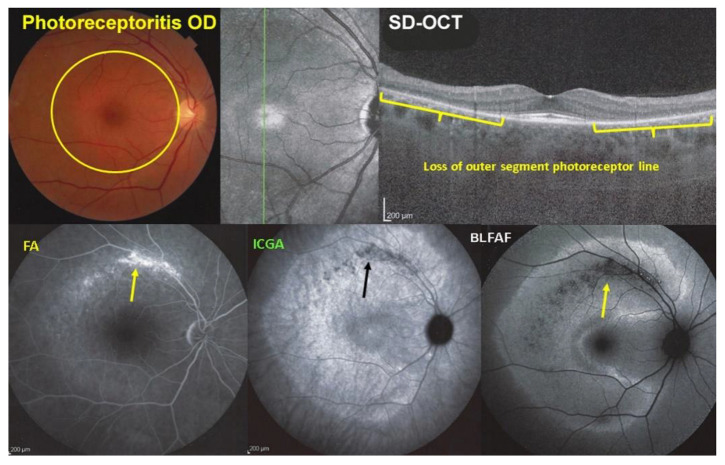
Multimodal Imaging of Photoreceptoritis case 2 (see text).

This 56-year-old woman was sent for a second opinion because of a visual field loss (MD = 6.2 dB) with a conserved BCVA of 1.0. Multimodal imaging is illustrated in Figure 11 and showed a pale discoloured halo around the normally coloured fovea (top left, yellow circle) explained by the loss of photoreceptor photopigment. The FA (bottom left picture) showed the same halo of discreet hyperfluorescence due to photopigment loss. An area of bright hyperfluorescence (window defect) along the superior temporal arcade due to chorioretinal atrophy was noted, dark on ICGA (bottom middle), and hypoautofluorescent on BL-FAF due to loss of RPE cells (bottom right). The ICGA (bottom middle) showed no hypofluorescent areas of choriocapillaris non-perfusion indicating preserved choriocapillaris (except in the arciform area of chorioretinal atrophy along the superior temporal arcade), with increased fluorescence in the area of loss of the screen of photopigments, which also explained the BL-FAF hyperautofluorescence (bottom right). The SD-OCT (top right) showed extensive loss of photoreceptor outer segments corresponding to the BL-FAF hyperautofluorescent area.

Photoreceptoritis case 3

This 46-year-old man complained of a sudden complete visual loss in his right eye. His vision was reduced to hand movements and he presented a severe visual field defect (MD = 19.6 dB).

**Figure 12:**
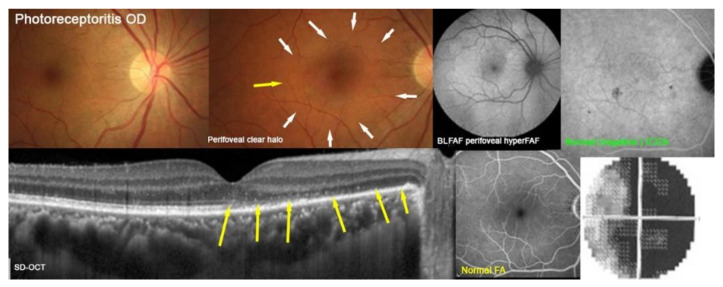
Multimodal Imaging of Photoreceptoritis case 3 (see text).

Multimodal imaging illustrates the lesions in Figure 12. Fundus photography showed a pale halo around the central macula (top two left pictures, white arrows). The BL-FAF showed diffuse hyperautofluorescence of the whole posterior pole except the central macula (top middle-right), and no ICGA hypofluorescent dots indicating an absence of choriocapillaris non-perfusion and a conserved choriocapillaris (top right) that could be seen. The SD-OCT (bottom left) showed a loss of photoreceptor outer segments corresponding to the BL-FAF hyperautofluorescent areas. The FA (bottom middle-right) was normal. The bottom right picture showed the extensive scotoma (MD = 19.6 dB).

Photoreceptoritis 4 and 5

This 44-year-old woman was sent to us for second advice by her uveitis specialist. She had consulted for bilateral photopsias, decreased contrast sensitivity, photophobia, and scotomas without any overt intraocular inflammation. At presentation, the BCVA was 0.2 (OD) and 0.4 (OS). Her visual fields were tubular (MD OD = 21.7 dB and MD OS = 21 dB).

**Figure 13:**
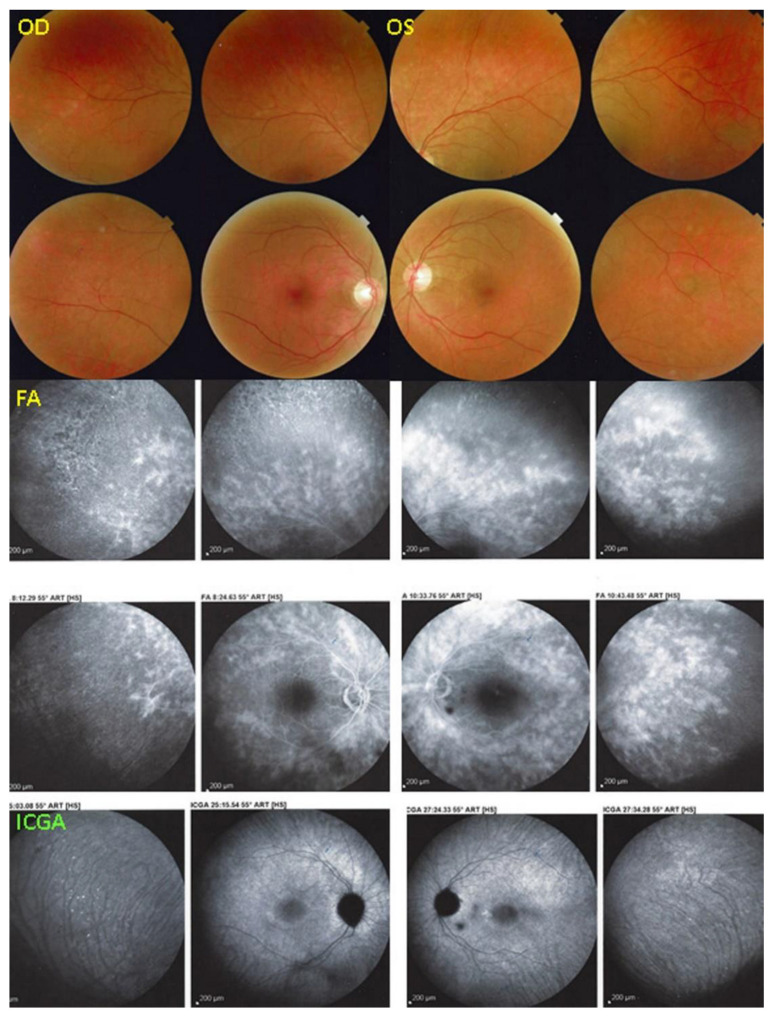
Photoreceptoritis case 4. Fundus examination (2 top quartets) showed faint discoloured rings OU around the central maculae. FA (middle quartets) showed bilateral retinal vasculitis. ICGA (bottom two pictures) no hypofluorescent dots were detected, indicating intact choriocapillaris circulation, except for two inter-papillo-macular dark atrophic dots OS.

**Figure 14:**
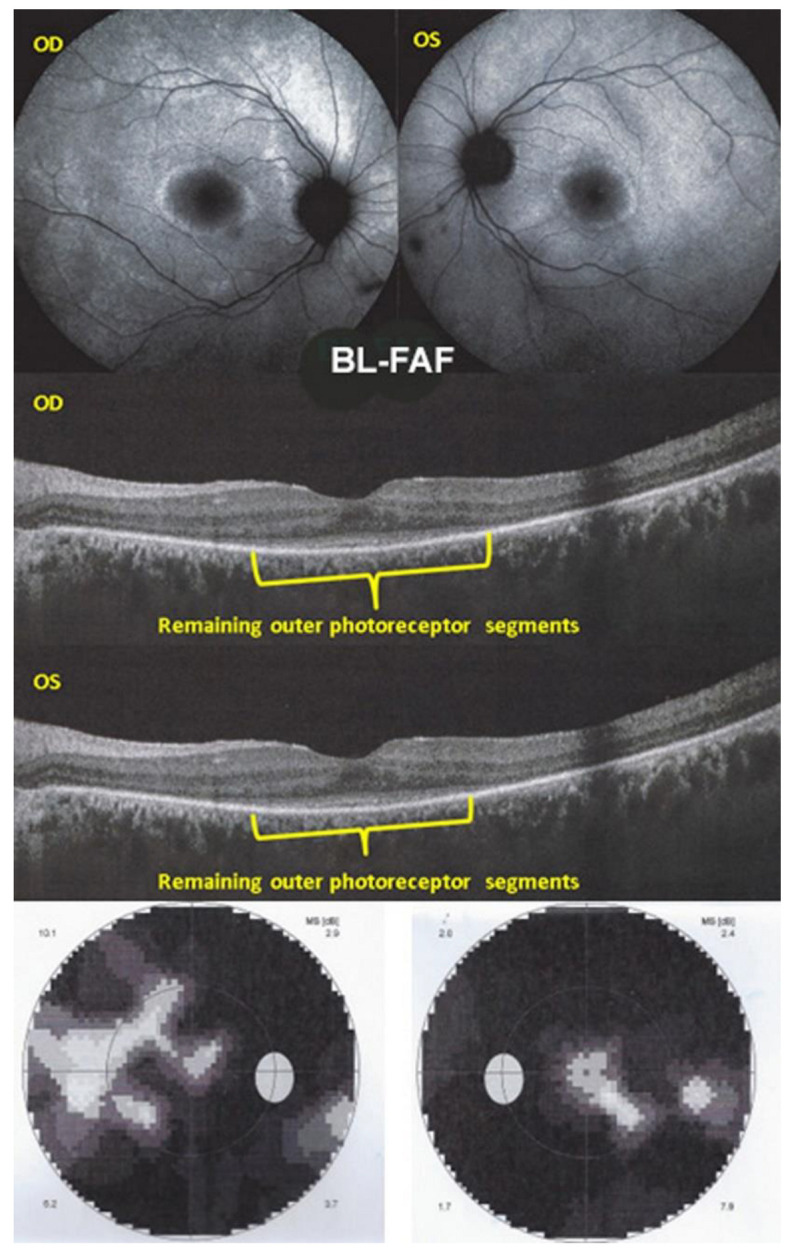
Multimodal Imaging of Photoreceptoritis case 4. SD-OCT (middle two pictures) showed a loss and/or disruption of the photoreceptor outer segment layer OU. BL-FAF (top two pictures) showed bilateral hyperautofluorescence in the posterior pole except in the central maculae OU. Severe visual field impairments OU were noted (two bottom pictures).

Fundus examination showed faint discoloured rings OU around the central maculae (Figure 13, 2 top quartets). The FA showed bilateral retinal vasculitis (Figure 12, middle quartets). The SD-OCT showed a loss and/or disruption of the photoreceptor outer segment layer OU (Figure 14, middle two pictures). The ICGA (Figure 13, bottom two pictures) was normal with no hypofluorescent dots, indicating intact choriocapillaris circulation, except for two inter-papillo-macular dark atrophic dots OS. The BL-FAF (Figure 14, top two pictures) showed bilateral hyperautofluorescence in the posterior pole except in the central maculae OU. Severe visual field impairments OU were noted (two bottom pictures of Figure 14). Noteworthy, in this case, was the BL-FAF bright hyperfluorescent ring between the normal central area and the surrounding diseased areas in both eyes. (Figure 14, top two images).

## 4. Summary of MMI Findings in Choriocapillaritis and Primary Photoreceptoritis

The MMI findings in eight cases of choriocapillaritis, including five MEWDS and three MFC patients, compared to three primary photoreceptoritis patients (four eyes) are summarised in Table 1.

**Figure 15:**
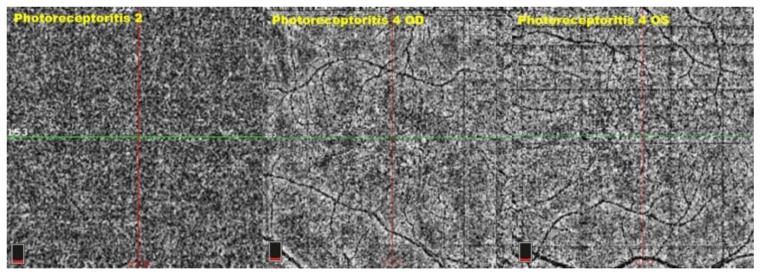
OCT-A in photoreceptoritis: three cases showing absence of choriocapillary drop-out.

Fundus findings were all characteristic of their respective diseases: white dots or foveal granularity for MEWDS, punched-out small scars for MFC, and perifoveal pale fundus halos for four out of the five cases of photoreceptoritis eyes, which was interpreted as annular loss of photopigment due to the loss of photoreceptor outer segments. The BL-FAF hyperautofluorescence was found in all 13 eyes together with loss and/or damage of IS/OS line on the SD-OCT, indicating a loss of photoreceptor outer segments in all cases. Most of our photoreceptoritis cases presented the characteristic tri-zonal pattern in the zone of transition from the healthy to the diseased area, shown in SD-OCT and BL-FAF; some studies presented this as a demarcating line of the progression at the level of the outer retina and a trizonal pattern of sequential involvement of the outer retina, retinal pigment epithelium, and choroid [12]. The only but important discrepancy between the MEWDS/MFC and the photoreceptoritis groups was the hypofluorescent areas on the ICGA, indicating choriocapillaris non-perfusion in the former group, which was absent in the latter group. Finally, the OCT-A showed an absence of choriocapillary drop-out in both groups with different explanations. In the choriocapillaritis group, the absence of OCT-A signs in the four cases where it was performed can be explained by the fact that end-choriocapillary non-perfusion cannot be detected by OCT-A in these low-flow vessels; in the photoreceptoritis group, there is absolutely no choriocapillaris non-perfusion (Figure 15). The FA signs were very variable from one case to the other in both groups, from an absence of significant signs to retinal hyperfluorescence and/or vasculitis, and did not contribute essentially to the explanation of the lesion processes. There was no significant difference in the BCVA among both groups (choriocapillaritis = 0.74 ± 0.28 versus 0.48 ± 0.4 in photoreceptoritis, *p* = 0.13, Student’s *t*-test), but visual field loss was significantly more important in the photoreceptoritis group with a mean of MD of 17.64 ± 6.45 dB versus 5.27 ± 3.9 dB in the choriocapillaritis group (*p* < 0.004, Student’s *t*-test). In the photoreceptoritis patient of case No. 4, both eyes showed a bright hyperautofluorescent ring around the central normal area, possibly indicating disease activity causing increased production and/or decreased clearance of lipofuscin by the RPE [13].

## 5. Discussion

Inflammatory damage to the outer retina has been ill-understood and classified indistinctly in the same pathology group, as thought by many including JD Gass [8,9,10,11]. Since MMI was developed in the mid-nineties of the last century, a finer analysis of these disorders has been possible.

The choriocapillaritis entities have been well-defined, although some divergent views still exist as far as MEWDS is concerned [4,14,15,16,17,18]. These disorders are generated by choriocapillaris non-perfusion of diverse severity and extension depending on the size of the choriocapillaris vessels involved in the process [4]. For MEWDS, small end-capillary low-flow vessels are involved, not detected by OCT-A, except for more pronounced cases that can produce choriocapillary drop-out [19]. MFC presents in the same fashion, although lesions are more extended producing small punched-out chorioretinal scars. Indeed, the first episode of MFC can be mistaken for MEWDS [20]. The sequence of events and the role of ischaemia has been explored experimentally in detail as explained in the introduction, and impaired glucose uptake in photoreceptors, due to ischaemia or other reasons, hampers the outer segment renewal and rod photoreceptor survival [1,2,21].

On the other hand, primary photoreceptoritis is due, at least for a substantial proportion of cases, to inflammatory causes, as several articles report the efficacy of corticosteroids and/or immunosuppressants in their treatment [6,22,23,24]. The mechanism consists of a direct insult to the photoreceptor. AZOOR typically is a condition producing primary photoreceptoritis [25].

Unfortunately, nowadays, many MMI studies do not include ICGA anymore, the only modality able to detect choriocapillary non-perfusion including the small low-flow end-capillary choriocapillaris vessels; therefore, differentiation between choriocapillaritis and photoreceptoritis is not possible. This is also the reason why MEWDS has been falsely interpreted as a photoreceptoritis [26,27,28,29]. The similar pathophysiology of MEWDS and MFC speaks to a common choriocapillaris-induced mechanism. Moreover, cases can present first as MEWDS and later when recurrences occur with small punched-out chorioretinal scars, should be re-diagnosed as MFC. Our second MFC case is an illustrative example of this phenomenon, which speaks to a common mechanism [20].

Although the mechanisms in choriocapillaritis and primary photoreceptoritis fundamentally differ, the occurrence of both conditions in the same patient has been described [20,23,30,31,32].

Usually, neither mild choriocapillaritis nor photoreceptoritis fatally damages the RPE cells, which are metabolically resistant [2]. For instance, the RPE is rarely damaged in MEWDS, in which the choriocapillaritis ischaemic intensity and durations are limited. In the case of MFC, the extension and intensity of ischaemia are more important, causing damage to the RPE cells observed as punched-out chorioretinal scars in cases of insufficient or delayed treatment. More aggressive choriocapillaritis entities, such as APMPPE/AMIC or serpiginous choroiditis, produce severe ischaemia leading to the loss of the usually metabolically resistant RPE cells, resulting in chorioretinal scars. Although primary photoreceptoritis is localised at the level of the photoreceptors, the disease can sometimes evolve towards chorioretinal atrophy and the loss of RPE cells, as shown in the second photoreceptoritis case, an evolution which has been termed as collateral damage by some authors [33].

The functional consequences, especially in visual field defects, are much more substantial in photoreceptoritis compared to choriocapillaritis; this can be explained by the fact that the photoreceptors are not directly the aim of the pathological process, but are only secondarily involved following perfusion problems of the choriocapillaris. Moreover, when looking at the literature, corticosteroid/immunosuppressive therapy seems to be more efficient in choriocapillaritis (for those conditions that need therapy) than in photoreceptoritis, where mixed results have been reported [34,35].

In summary, MMI allows us to differentiate more clearly the pathophysiology of outer retinal diseases. The determining imaging modality that allows us to distinguish between photoreceptoritis and choriocapillaritis is ICGA.

## Figures and Tables

**Table 1:** Multimodal Imaging findings of patients with MEWDS, MFC compared to patients with Photoreceptoritis.

	Fundus	BL-FAF	ICGA	SD-OCT	FA	OCT-A
MEWDS (*n* = 5)	Foveal granularity 4/5	Positive-hyperauto-FAF 5/5	Positive-hypofluo 5/5	Damage/loss of IS/OS 5/5	no/faint hyperfluor 1/5	negative 2/2
		White dots 1/5				moderate hyperfluor 4/5
MFC (*n* = 3)	Punched-out small scars	Positive-hyperauto-FAF 3/3	Positive-hypofluo 3/3	Damage/loss of IS/OS 3/3	no/faint hyperfluor 3/3	negative 3/3
Photoreceptoritis (*n* = 5)	Pale halo around fovea 4/5	Positive-hyperauto-FAF 3/3	**Negative-no-hypofluo 5/5**	Damage/loss of IS/OS 5/5	no hyperfluor 3/3	negative 3/3
				**No non-perfusion**		moderate hyperfluor 2/2

BL-FAF: Blue light autofluorescence, ICGA: Indocyanine angiography, SD-OCT: Spectral-domain optical coherence tomography, FA: Fluoresceine angiography, OCT-A: OCT angiography, MEWDS: Multiple evanescent white dots syndrome, IS/OS: Inner segment/outer segment, MFC: Multifocal choroiditis.

## Data Availability

The data presented in this study are available on request from the corresponding author. The data are not publicly available due to privacy constraints.
